# Personal exposure to fine particulate air pollutants impacts blood pressure and heart rate variability

**DOI:** 10.1038/s41598-020-73205-x

**Published:** 2020-10-06

**Authors:** Dong-Hoon Lee, Sun-Hwa Kim, Si-Hyuck Kang, Oh Kyung Kwon, Jin-Joo Park, Chang-Hwan Yoon, Young-Seok Cho, Jongbae Heo, Seung-Muk Yi, Tae-Jin Youn, In-Ho Chae

**Affiliations:** 1 Department of Internal Medicine, Cardiovascular Center, Seoul National University Bundang Hospital, 82, Gumi-Ro 173 Beon-Gil, Bundang-Gu, Seongnam-Si, Gyeonggi-Do South Korea; 2 Department of Internal Medicine, Seoul National University, Seoul, South Korea; 3 Busan Development Institute, Busan, South Korea; 4 Department of Environmental Health and Institute of Health and Environment, Graduate School of Public Health, Seoul National University, Seoul, South Korea

**Keywords:** Epidemiology, Arrhythmias, Hypertension, Arrhythmias, Hypertension

## Abstract

Air pollution has increasingly been recognized as a major healthcare concern. Air pollution, particularly fine particulate matter (≤ 2.5 μm in aerodynamic diameter [PM_2.5_]) has demonstrated an increase in adverse cardiovascular events. This study aimed to assess the cardiovascular response to personal exposure to different levels of PM_2.5_. This prospective cohort study enrolled healthy volunteers aged ≥ 18 years with no cardiovascular disease. Study subjects carried personal exposure monitor of PM_2.5_, digital thermo-hygrometer for temperature and humidity, 24-h blood pressure monitor, and continuous electrocardiogram monitor. Measurements were repeated twice with an interval of 6–12 months. Statistical models consisted of generalized estimation equations to various repeated measures of each subject. A total of 22 subjects were enrolled in this study between July 2018 and January 2019. Measurement was performed twice in all participants, and a total of 36 data were collected except for insufficient data collection. The mean age of the study population was 41.6 years, and 95% of the subjects were females. No study subjects had hypertension or other cardiovascular diseases. The average systolic blood pressure increased with higher PM_2.5_ levels with marginal significance (0.22 mmHg [95% confidential intervals − 0.04 to 0.48 mmHg] per 10 μg/m^3^ of PM_2.5_). All parameters for heart rate variability significantly decreased with a higher level of PM_2.5_. In this study, we measured individual personal exposure to PM_2.5_ by using a portable device. We found that 24-h exposure to high levels of PM_2.5_ was associated with a significant decrease in heart rate variability, suggesting impaired autonomous nervous function.

## Introduction

Exposure to air pollution increases mortality and morbidity. According to the World Health Organization, ambient and household air pollution are responsible for 7.6% and 7.7% of the global mortality, respectively^1^. Exposure to ambient fine particulate matter with an aerodynamic diameter of < 2.5 μm (PM_2.5_) has been considered as the most important environmental health hazard. The Global Burden of Diseases Study estimated ambient PM_2.5_ as the fifth most important risk factor for mortality worldwide^2^. Various human activities, including use of motor vehicles, industrialization, and biomass combustion have been associated with increased levels of outdoor air pollution.

Exposure to air pollution also occurs from indoor environments. People in industrialized countries are reported to spend approximately 90% of their time indoors^3^. Solid fuels used in cooking or heating, poor ventilation, and tobacco products are the major contributors to indoor air pollution in developing countries. In more developed parts of the world, air conditioning systems, formaldehyde exposure, and exposure to second-hand cigarette smoke contribute to increased levels of indoor air pollution^4^.

Studies have linked short-term exposure to PM_2.5_ with cardiovascular death and diseases^5^. Exposure to particulate matter (PM) air pollution has been shown to decrease vagal tone, resulting in decreased heart rate variability (HRV)^6^. However, BP response to short-term exposure to PM_2.5_ has been inconclusive.

There have been limited studies that monitored individual levels of PM_2.5_ and assessed cardiovascular parameters in routine environments of study subjects. In this study, we estimated the personal exposure to PM_2.5_ using portable particle counters. Cardiovascular response of the study subjects, including BP and electrocardiogram (ECG) were collected using 24-h monitor devices.

## Methods

### Design

This prospective study enrolled healthy adults aged ≥ 18 years without cardiovascular disease. Volunteers were recruited using poster advertisements from the Seoul National University Bundang Hospital, Korea from July 2018 to January 2019. The exclusion criteria included (1) medical treatment that may modify study measurements, such as BP-lowering agents, antiarrhythmics, and beta-blocker, (2) cognitive impairment, such as dementia, delirium, and amnestic disorders that may cause difficulty in study device management, and (3) a serious or unstable medical or psychological condition that is judged to be unable to successfully participate in the research at the researcher's discretion. Study participants were recommended to repeat study measurements twice with an interval of 6 months (a window period from 6 to 12 months). The study protocol was approved by the Seoul National University Bundang Hospital Institutional Review Board (protocol number: B-1810-499-307) and experiments were conducted according to the Helsinki Declaration (Revised in 2013) and ICH-GCP. The participants provided written informed consent.

### Measurements

**Figure 1:**
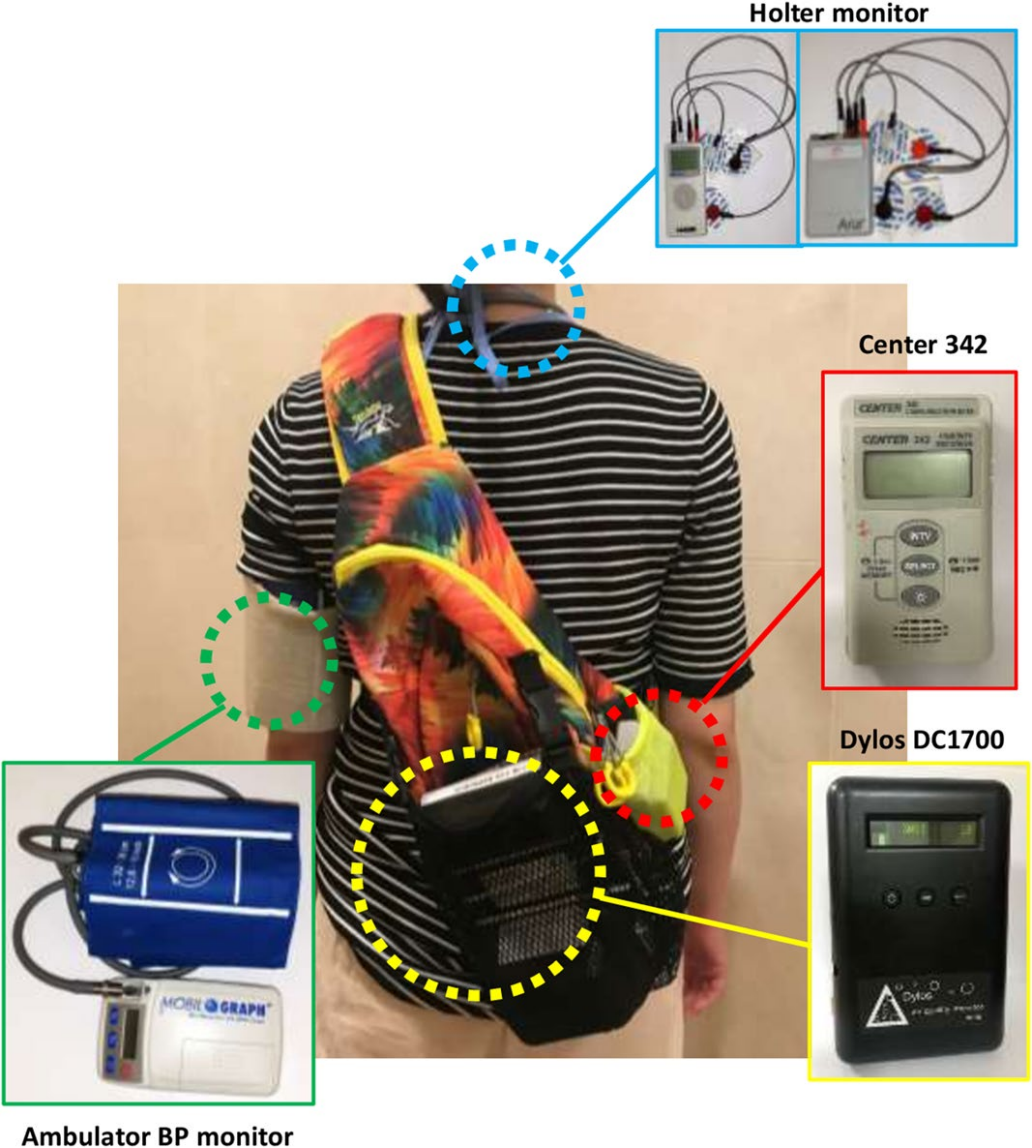
Study devices for the study. A study subject carries four study devices for 24 h: Dylos DC1700 for PM_2.5_ monitor, Center 342 to monitor the temperature and humidity, Holter monitor, and an ambulatory blood pressure monitor.

Participants were recommended to perform his/her daily routine while carrying four devices: personal air quality monitor, temperature/humidity data logger, ambulator BP monitor, and 24-h ECG monitor. Personal exposure to PM was measured using Dylos DC1700 (Dylos Corporation, CA, USA), which is a battery-operated particle counter based on light-scattering technology. Its performance has been validated in previous studies^7,8^ (Fig. 1). Personal exposure to PM_2.5_ was calculated as described previously^9^

$${\text{Exposed PM}}_{2.5} = 0.65 + 4.16\times10^{-5} \left({\text{measured particle}}\right) + 1.57\times 10^{-11} \left({\text{measured particle}}\right)^{2}$$

Center 342 (Center Technology Corp, Taiwan), a portable data logger, was used to record temperature and humidity. Ambulatory BP monitor (Mobil-O Graph, IEM, Germany), and continuous ECG monitor (Aria recorder, Del Mar Reynolds Medical Ltd., UK and EVO, Spacelabs Healthcare, US) were also used. The average, daytime, and nighttime, a ratio of a dipper, and standard deviation (SD) of systolic and diastolic BP were driven from the measurements of the ambulatory BP monitors. Heart rate, percentage of ventricular and supraventricular premature beats and parameters for heart rate variability were calculated from the continuous ECG monitoring. Heart rate variability was measured using time domain methods^6,10^. The measured variables included standard deviation of NN interval (SDNN), root mean square of successive differences (rMSSD), standard deviation of successive differences (SDSD), HRV index, triangular interpolation of NN interval histogram (TINN), and the proportion of the number of pairs of successive NNs that differ by more than 50 ms (pNN50).

### Statistical analysis

Continuous variables are reported as mean ± standard deviation or median (interquartile ranges) as appropriate. Categorical variables are reported as number (proportion). Statistical models were constructed with generalized estimating equations with the variable number of repeated measures on each subject. The main independent variable was daily mean personal exposure to PM_2.5_, while covariates included mean temperature, humidity, age, and sex. The dependent variables included average, daytime, nighttime, the ratio of a dipper, and standard deviation of systolic and diastolic BP, heart rate, percentage of ventricular and supraventricular premature beats, and parameters for HRV. Sensitivity analyses were performed to test the robustness of the results: first testing study seasons as a confounder, and second applying spline to temperature and humidity. Statistical analyses were performed using the R programming version 3.6.1 (The R Foundation for Statistical Computing, Vienna, Austria; https://www.R-project.org). A two-sided p-value < 0.05 was considered statistically significant.

## Results

**Figure 2:**
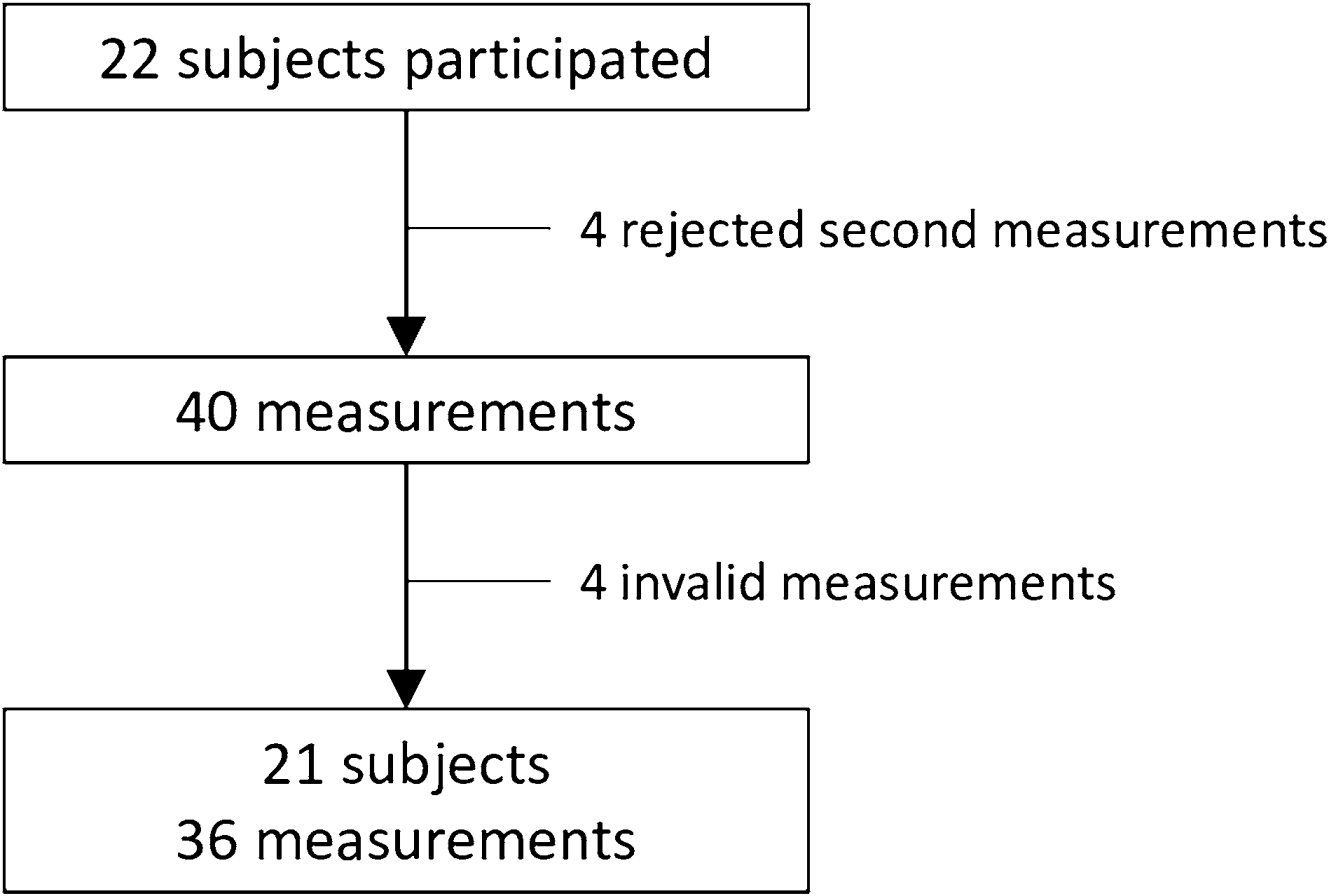
Flow chart of study patients and measurements.

Twenty-two adults voluntarily participated in the study. The study protocol mandated two repeated measurements with an interval of 6 months. Four participants refused the second set of measurements, and a total of 40 measurements were collected. Most participants (34 out of 40) started monitoring in the afternoon and finished the next morning. Four measurements were excluded owing to insufficient data collection. Therefore, 36 measurements from the 21 study subjects were collected and analyzed in the study (Fig. 2). Table 1 shows the general characteristics of the participants: 20 of whom were females, and the mean age was 41.6 ± 11.1 (ranges 25‒55) years. No subjects had a history of cardiovascular disease.

**Table 1:** Baseline characteristics of participants.

	Mean (SD) or number (%)
**All**	21 (100%)
**Age (years)**	41.6 (25–55)
18–29	5
30–49	8
≥ 50	8
**Sex**	
Male	1 (5%)
Female	20 (95%)
**Underlying disease**	
Hypertension	0 (0%)
Arrhythmia	0 (0%)
Heart failure	0 (0%)
Coronary artery disease	0 (0%)
Current asthma	2(9%)
**Systolic BP (mmHg)**	
Average	110.6 (6.64)
Day	112.4 (6.45)
Night	106.7 (9.99)
Dipper	5.08 (6.55)
SD day	11.14 (3.07)
SD night	7.06 (2.93)
**Diastolic BP (mmHg)**	
Average	68.83 (6.72)
Day	70.28 (6.73)
Night	65.58 (8.18)
Dipper	6.62 (8.03)
SD day	8.96 (3.01)
SD night	6.39 (2.23)
**Heart rate (bpm)**	
Min	54.56 (9.27)
Max	135.0 (17.63)
Average	76.31 (12.11)
**Premature beat**	
SVPB (%)	9.44 (21.93)
VPB (%)	2.28 (6.24)
**Heart rate variability**	
SDNN total (ms)	150.6 (40.47)
rMSSD total (ms)	37.29 (15.13)
SDSD total (ms)	27.01 (11.89)
HRV index (ms)	22.44 (10.44)
TINN (ms)	627.8 (207.20)
pNN50 total (%)	10.93 (9.10)

Values are shown as mean (SD) or n (%).

*BP* blood pressure, *HRV* heart rate variability, *pNN50* proportion of the number of pairs of successive normal-to-normal that differ by more than 50 ms, *rMSSD* root mean square of successive differences, *SD* standard deviation, *SDNN* standard deviation of normal-to normal interval, *SDSD* standard deviation of successive differences, *SVPB* supraventricular premature beat, *TINN* triangular interpolation of normal-to-normal interval histogram, *VPB* ventricular premature beat.

The mean temperature during which the study subjects performed measurements was 24.6 ± 2.6 °C. During the same period, the average PM_2.5_ measured by personal exposure monitor was 20.4 ± 51.6 μg/m^3^ (Table 2) (Supplementary Fig. 1). Correlations between air pollutants, temperature, and relative humidity are detailed in Supplementary Table 1.

**Table 2:** Summary statistics of measured meteorological and air pollution data.

	Daily mean (SD)	Quantiles					Interquartile range
		Min	25%	50%	75%	Max	
PM_2.5_ (μg/m^3^)	20.4 (51.9)	1.8	4.5	7.2	15.2	313.9	10.7
Temperature (°C)	24.6 (2.6)	19.7	22.2	24.8	26.0	29.4	3.8
Humidity (%RH)	47.1 (13.9)	19.9	37.8	47.5	58.9	70.3	21.1

*PM*_*2.5*_ fine particulate matter (< 2.5 μm), *RH* relative humidity.

Parameters measured by the Holter monitor and ambulatory BP monitor are summarized in Table 3. The mean BP was 110.6/68.9 mmHg, and the mean heart rate was 76.3 ± 12.1 per min. Correlation matrix of measured values is shown Supplementary Table 2. Holter and ambulator BP monitor parameters were analyzed in relation with personal exposure to PM_2.5_ after adjusting for age, sex, mean temperature, and mean humidity. Average systolic BP tended to increase with higher PM2.5 levels with marginal significance (P = 0.098). Otherwise, none of the 24-h systolic and diastolic BP components showed significant association with PM_2.5_ levels. Heart rate and frequencies of ventricular and supraventricular ectopic beats also showed no significant relationship with PM_2.5_. Individual exposure to PM_2.5_ showed a significant relationship with HRV. All parameters used for heart rate variability (SDNN, rMSSD, SDSD, HRV index, TINN, and pNN50) were shown to decrease with higher levels of PM_2.5_. Sensitivity analyses confirmed that the study season had no significant confounding effects and that applying spline for independent variables did not change the results remarkably.

**Table 3:** Estimated effects of air pollution on measured variables.

Outcome variables	Unadjusted		Adjusted	
	Coefficient (95% CI)	P-value	Coefficient (95% CI)	P-value
**Systolic BP (mmHg)**				
Average	0.17 (− 0.04 to 0.37)	0.106	0.22 (− 0.04 to 0.48)	0.098
Day	0.11 (− 0.07 to 0.30)	0.223	0.19 (− 0.06 to 0.43)	0.144
Night	0.29 (0.01 to 0.57)	0.039	0.30 (− 0.08 to 0.69)	0.124
Dipper	− 0.16 (− 0.31 to − 0.01)	0.034	− 0.11 (− 0.37 to 0.15)	0.411
SD day	− 0.03 (− 0.08 to 0.03)	0.334	0.03 (− 0.06 to 0.11)	0.548
SD night	− 0.03 (− 0.08 to 0.02)	0.238	0.02 (− 0.07 to 0.11)	0.654
**Diastolic BP (mmHg)**				
Average	0.01 (− 0.16 to 0.19)	0.880	0.06 (− 0.15 to 0.27)	0.560
Day	0.07 (− 0.11 to 0.24)	0.445	0.10 (− 0.08 to 0.28)	0.293
Night	− 0.09 (− 0.30 to 0.13)	0.436	0.01 (− 0.34 to 0.35)	0.976
Dipper	0.20 (0 to 0.40)	0.049	0.10 (− 0.25 to 0.46)	0.568
SD day	− 0.09 (− 0.14 to − 0.03)	0.002	− 0.03 (− 0.10 to 0.05)	0.486
SD night	0.06 (0 to 0.11)	0.036	0.08 (0.00 to 0.17)	0.052
**Heart rate (bpm)**				
Min	0.21 (− 0.09 to 0.52)	0.170	0.14 (− 0.30 to 0.58)	0.545
Max	0.41 (− 0.01 to 0.84)	0.056	0.09 (− 0.47 to 0.64)	0.761
Average	− 0.06 (− 0.50 to 0.37)	0.778	− 0.21 (− 0.81 to 0.40)	0.509
**Premature beat**				
SVPB (%)	0.07 (− 0.67 to 0.80)	0.861	0.23(− 0.45 to 0.92)	0.506
VPB (%)	− 0.01 (− 0.15 to 0.12)	0.851	− 0.01 (− 0.16 to 0.13)	0.866
**Heart rate variability**				
SDNN total (ms)	− 1.56 (− 2.67 to − 0.45)	0.006	− 1.94 (− 3.59 to − 0.29)	0.021
rMSSD total (ms)	− 0.45 (− 0.78 to − 0.13)	0.006	− 0.66 (− 1.19 to − 0.13)	0.015
SDSD total (ms)	− 0.36 (− 0.60 to − 0.13)	0.003	− 0.53 (− 0.90 to − 0.15)	0.006
HRV index (ms)	− 0.33 (− 0.59 to − 0.07)	0.014	− 0.32 (− 0.61 to − 0.03)	0.028
TINN (ms)	− 6.20 (− 11.74 to − 0.66)	0.028	− 8.98 (− 17.46 to − 0.49)	0.038
pNN50 total (%)	− 0.31 (− 0.51 to − 0.11)	0.003	− 0.46 (− 0.81 to − 0.11)	0.009

Estimated effects and 95% confident intervals (CI) for each change of PM_2.5_ by 10 μg/m^3^.

Unadjusted and adjusted coefficients for sex, age, temperature and humidity.

*BP* blood pressure, *CI* confidence interval, *HRV* heart rate variability, *pNN50* proportion of the number of pairs of successive normal-to-normal that differ by more than 50 ms, *rMSSD* root mean square of successive differences, *SD* standard deviation, *SDNN* standard deviation of normal-to normal interval, *SDSD* standard deviation of successive differences, *SVPB* supraventricular premature beat, *TINN* triangular interpolation of normal-to-normal interval histogram, *VPB* ventricular premature beat.

## Discussion

This study demonstrates the cardiovascular response to personal exposure to PM_2.5_ in healthy adults with no cardiovascular disease. We found no significant effects on BP either during the daytime or night-time. However, personal exposure to a higher level of PM_2.5_ was significantly associated with a decrease in HRV indicating impaired response of the autonomous nervous system.

Strong evidence supports the impact of long-term and short-term exposure to ambient air pollution on mortality^11,12^. Ambient air pollution has also been linked with a number of cardiovascular diseases^5,13,14^. Most of the previous studies regarded outdoor air pollution levels as exposure variables. However, a subject’s exposure to air pollution also originates from various sources as people stay indoors most of the day in modern societies^3^. Studies with indoor air pollution have been sparse, and this could be attributed to the difficulty in quantitative measurement of long-term individual exposure from the environment.

This study measured 24-h individual PM_2.5_ levels by using a portable personal monitor. In addition, study subjects repeated the same measurements 6 months later to consider inter- and intra-individual variations. Individual exposure as measured by portable devices showed large fluctuations depending on the subjects’ situations. In general, the levels showed high correlation with the ambient PM_2.5_ levels measured at the nearby local monitoring station on the study dates (Supplementary Table 3). However, it showed larger variations during the day as some subjects showed high levels during transportation and some showed low or high levels at home.

The cardiovascular responses to indoor and outdoor air pollution continuously measured using personal monitors in this study were in line with those seen in previous studies. There was a marginal trend toward positive relationship between PM_2.5_ levels and average systolic BP. However, the effect estimate was small (0.22 mmHg per 10 μg/m^3^), and other parameters from ambulator BP monitors showed no significant relationships. Previous studies have shown conflicting results for short-term relationship between particulate air pollutants and arterial BP^15–18^. One possible explanation is the lag effect. Positive relationships shown in the previous studies were seen at 1‒5 lag days. In the present study, all the 24-h measurements were performed during a single day. Another issue is the study population. Many of the studies showing positive relationship enrolled patients who already had hypertension or cardiovascular disease. Hypertensive adults generally have higher BP variability than normotensive ones^19^. They may be more sensitive to environmental factors, such as air pollution than healthy young subjects without cardiovascular diseases, who comprised the study population in the present study.

We also found that autonomic nervous system dysfunction was associated with response to high levels of PM_2.5_. The association between particulate air pollutants and reduced HRV has been shown in a number of studies^6,10,20^. This study also showed that HRV parameters assessed by time domain methods significantly decreased with PM_2.5_ levels. HRV is a marker used for evaluation of cardiac autonomic function. Studies have suggested that reduced HRV is associated with increased risk of cardiovascular events^21^. Suggested mechanisms include disturbances in cardiac autonomic control, reduction in cardiac vagal control, decreases in parasympathetic tone, and an imbalance in cardiac autonomic control^22–24^.

This study has several limitations. First, as healthy adults without cardiovascular disease were enrolled, the study findings cannot be extrapolated to the general population. Second, average of PM_2.5_ for 24 h was analyzed using daily parameters of cardiovascular markers. Short-term responses, such as hour lag models were not fully addressed in this study. Third, this study was principally observational and other factors that may affect BP or heart rate could not be controlled. Environmental factors, such as physical activity and emotional stress, may also influence the cardiovascular system. In addition, strict control of study hours and weekdays could have improved the study quality. Fourth, several potential confounders such as smoking status and body mass index were not collected. Fifth, we cannot exclude the possibility of play of chance because we performed multiple testing simultaneously in this study. Lastly, information on other air pollutants, such as PM_10_ and gaseous pollutants were not assessed in this study. Short-term BP response to gaseous pollutants, such as ozone has been reported^25^.

## Conclusions

In this study, we analyzed the cardiovascular response including BP, heart rate, and HRV in response to personal exposure to PM_2.5_ in healthy adults. There was no significant impact of PM_2.5_ on systolic or diastolic blood pressure measured by ambulatory BP monitor for 24 h. However, exposure to high levels of PM_2.5_ was significantly associated with reduced HRV suggesting impaired autonomous nervous function.

## Supplementary information

Supplementary Information.
