# Supplementary material for: Personal exposure to fine particulate air pollutants impacts blood pressure and heart rate variability
Source: Sci Rep. 2020 Oct 6;10:16538. doi: 10.1038/s41598-020-73205-x (PMC7538889; doi:10.1038/s41598-020-73205-x)
Supplement: Supplementary file 1 — Supplementary Information. [file 41598_2020_73205_MOESM1_ESM.docx]

**Supplementary Information**

**Personal exposure to fine particulate air pollutants impacts blood pressure and heart rate variability**

Dong-Hoon Lee, MD^1,2^, Sun-Hwa Kim, PhD^1^, Si-Hyuck Kang, MD, PhD^1,2^, Oh Kyung Kwon, MD^1,2^, Jin-Joo Park, MD, PhD^1,2^, Chang-Hwan Yoon, MD, PhD^1,2^, Young-Seok Cho, MD^1,2^, Jongbae Heo, PhD^3^, Seung-Muk Yi, PhD ^4^, Tae-Jin Youn, MD^1,2^, In-Ho Chae, MD^1,2^

^1^Cardiovascular Center, Seoul National University Bundang Hospital, Seongnam-si, Korea; ^2^Department of Internal Medicine, Seoul National University, Seoul, Korea; ^3^Busan Development Institute; ^4^Department of Environmental Health and Institute of Health and Environment, Graduate School of Public Health, Seoul National University, Seoul, Korea

**Supplementary Table 1.** Correlation matrix among PM_2.5_, temperature and humidity

|  | **PM_2.5_** | **Temperature** | **Humidity** |
| --- | --- | --- | --- |
| **PM_2.5_** | - | -0.152 | 0.005 |
| **Temperature** |  | - | 0.176 |
| **Humidity** |  |  | - |

PM_2.5_; fine particulate matter (<2.5 μm)

**Supplementary Table 2.** Correlation matrix among blood pressure and heart rate variability parameters

|  | **Systolic BP** | **Diastolic BP** | **Heart Rate** | **SVPB** | **VPB** | **SDNN total** | **rMSSD total** | **SDSD total** | **HRV index** | **TINN** | **pNN50 total** |
| --- | --- | --- | --- | --- | --- | --- | --- | --- | --- | --- | --- |
| **Systolic BP** | - | 0.734 | 0.429 | 0.059 | 0.093 | -0.344 | -0.326 | -0.303 | -0.154 | -0.341 | -0.343 |
| **Diastolic BP** |  | - | 0.176 | 0.126 | 0.098 | -0.337 | -0.314 | -0.332 | 0.054 | -0.195 | -0.281 |
| **Heart Rate** |  |  | - | -0.124 | -0.002 | -0.621 | -0.450 | -0.346 | -0.440 | -0.598 | -0.485 |
| **SVPB** |  |  |  | - | 0.892 | 0.236 | 0.231 | 0.302 | 0.165 | 0.246 | 0.063 |
| **VPB** |  |  |  |  | - | 0.217 | 0.137 | 0.216 | 0.131 | 0.260 | -0.015 |
| **SDNN total** |  |  |  |  |  | - | 0.629 | 0.569 | 0.498 | 0.746 | 0.673 |
| **rMSSD total** |  |  |  |  |  |  | - | 0.979 | 0.200 | 0.390 | 0.925 |
| **SDSD total** |  |  |  |  |  |  |  | - | 0.141 | 0.311 | 0.841 |
| **HRV index** |  |  |  |  |  |  |  |  | - | 0.727 | 0.301 |
| **TINN** |  |  |  |  |  |  |  |  |  | - | 0.485 |
| **pNN50 total** |  |  |  |  |  |  |  |  |  |  | - |

BP = blood pressure; HRV = heart rate variability; pNN50 = proportion of the number of pairs of successive normal-to-normal that differ by more than 50 msec; rMSSD = root mean square of successive differences; SDNN =standard deviation of normal-to normal interval; SDSD = standard deviation of successive differences; SVPB = supraventricular premature beat; TINN = triangular interpolation of normal-to-normal interval histogram; VPB = ventricular premature beat

**Supplementary Table 3.** Summary statistics of data of the nearest measuring facility

|  | **Daily mean (SD)** | **Quantiles** | | | | | **Interquartile range** |
| --- | --- | --- | --- | --- | --- | --- | --- |
|  |  | **Min** | **25%** | **50%** | **75%** | **Max** |  |
| **PM_2.5_ (μg/m^3^)** | 22.29 (9.93) | 11.0 | 16.0 | 17.0 | 29.0 | 38.0 | 13.0 |
| **Temperature (°C)** | 13.70 (12.36) | -1.14 | 3.68 | 12.60 | 24.31 | 28.43 | 20.63 |
| **Humidity (%RH)** | 68.11 (7.53) | 56.16 | 64.61 | 71.18 | 72.50 | 75.22 | 7.89 |

PM_2.5_; fine particulate matter (<2.5 μm); RH = relative humidity

**Supplementary Figure 1.** Average of hourly measurements of PM_2.5_, temperature and humidity

**
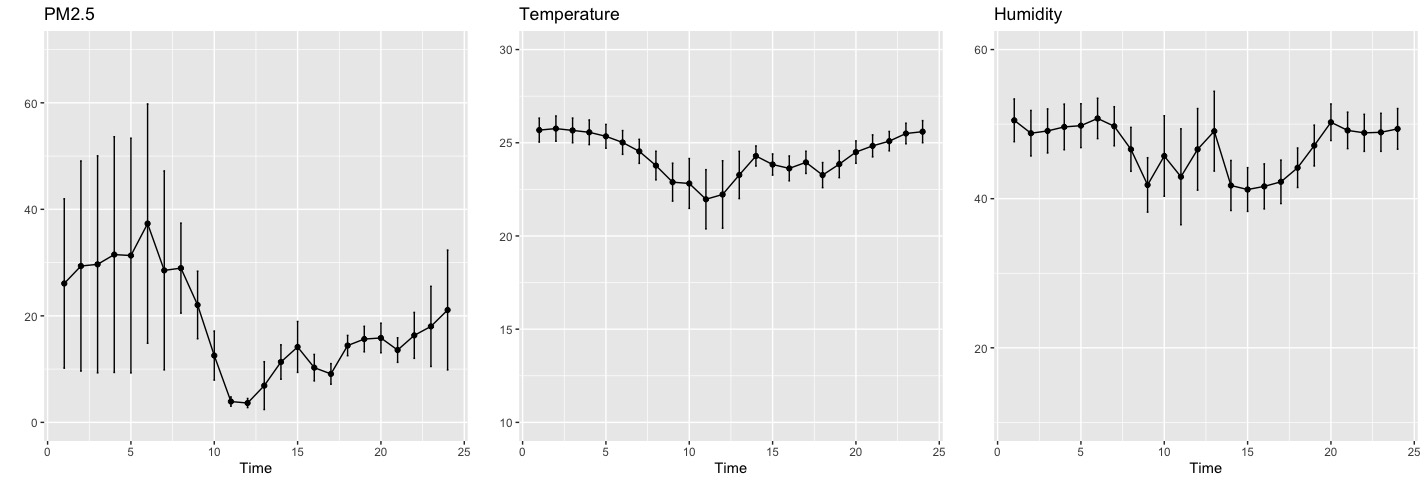
**

PM_2.5_; fine particulate matter (<2.5 μm) (μg/m^3^), Temperature (°C), Humidity (% relative humidity)
